# Synthesis and Validation of a Hydroxypyrone-Based, Potent, and Specific Matrix Metalloproteinase-12 Inhibitor with Anti-Inflammatory Activity *In Vitro* and *In Vivo*

**DOI:** 10.1155/2015/510679

**Published:** 2015-08-13

**Authors:** J. Aerts, R. E. Vandenbroucke, R. Dera, S. Balusu, E. Van Wonterghem, L. Moons, C. Libert, W. Dehaen, L. Arckens

**Affiliations:** ^1^Laboratory of Neuroplasticity and Neuroproteomics, KU Leuven, Naamsestraat 59, 3000 Leuven, Belgium; ^2^Inflammation Research Center, VIB, Technologiepark 927, 9052 Ghent, Belgium; ^3^Department of Biomedical Molecular Biology, Ghent University, Technologiepark 927, 9052 Ghent, Belgium; ^4^Molecular Design and Synthesis, KU Leuven, Celestijnenlaan 200f, 3001 Leuven, Belgium; ^5^Organic and Bio-Polymer Chemistry, UHasselt, Agoralaan Building D, 3590 Diepenbeek, Belgium; ^6^Laboratory of Neural Circuit Development and Regeneration, KU Leuven, Naamsestraat 61, 3000 Leuven, Belgium

## Abstract

A hydroxypyrone-based matrix metalloproteinase (MMP) inhibitor was synthesized and assayed for its inhibitory capacity towards a panel of ten different MMPs. The compound exhibited selective inhibition towards MMP-12. The effects of inhibition of MMP-12 on endotoxemia and inflammation-induced blood-cerebrospinal fluid barrier (BCSFB) disruption were assessed both *in vitro* and *in vivo*. Similar to MMP-12 deficient mice, inhibitor-treated mice displayed significantly lower lipopolysaccharide- (LPS-) induced lethality compared to vehicle treated controls. Following LPS injection *Mmp-12* mRNA expression was massively upregulated in choroid plexus tissue and a concomitant increase in BCSFB permeability was observed, which was restricted in inhibitor-treated mice. Moreover, an LPS-induced decrease in tight junction permeability of primary choroid plexus epithelial cells was attenuated by inhibitor application *in vitro*. Taken together, this hydroxypyrone-based inhibitor is selective towards MMP-12 and displays anti-inflammatory activity *in vitro* and *in vivo*.

## 1. Introduction

Matrix metalloproteinases (MMPs) are Zn^2+^-dependent endopeptidases that are implicated in numerous physiological processes such as reproduction, wound repair, development, and immune function as well as pathological conditions such as cancer, inflammation, neurodegenerative disorders, and autoimmune diseases [1–4]. The expression of many MMP family members is enhanced in endotoxemia [5–7] and cecal ligation and puncture (CLP) [8–10], two mouse models of sepsis, a highly mortal infectious disease, which still lacks an efficient therapeutic treatment. Additionally, studies using MMP inhibitors and MMP knockout mice indicate that MMPs play a detrimental role in sepsis [9, 11–13]. Moreover, several human data have directly correlated higher levels of active MMP with lower rates of survival in sepsis patients and some MMPs are considered as valuable biomarkers in sepsis [8, 9, 14, 15].

Their detrimental role in several inflammatory diseases justifies the development of specific MMP inhibitors, which have been intensely sought after for over two decades but with poor success [4, 16]. Most MMP inhibitors (MMPi) consist of two distinct features: a peptidomimetic backbone that interacts noncovalently with specific subsites of the catalytic domain that govern substrate interaction and a Zn^2+^-chelating moiety or zinc-binding group (ZBG) that binds to the hydrolytic Zn^2+^-ion via coordinate-covalent bonds, rendering the enzyme inactive [16, 17]. The majority of MMPi were initially synthesized containing hydroxamic acid as ZBG which, although very potent, resulted in poor specificity, oral availability, or biocompatibility [18]. To circumvent these problems, pyrone-based inhibitors emerged as superior MMPi with known biocompatibility, good aqueous solubility, and synthetic versatility [19, 20]. Based on this strategy, a specific MMP-3 pyrone-based inhibitor (AM-6) was developed [21]. However, the specificity of AM-6 was only characterized for MMP-1, MMP-2, and MMP-3 and therefore, in this study, we synthesized the same compound and screened it against a broader range of MMPs to firmly ascertain MMP selectivity. Surprisingly, the compound displayed specificity and potency towards MMP-12. To accommodate these findings, we show that the inhibitor protects against inflammation-induced disruption of the blood-cerebrospinal fluid barrier both* in vitro* and* in vivo*.

## 2. Materials and Methods

### 2.1. Chemistry

**Scheme 1:**
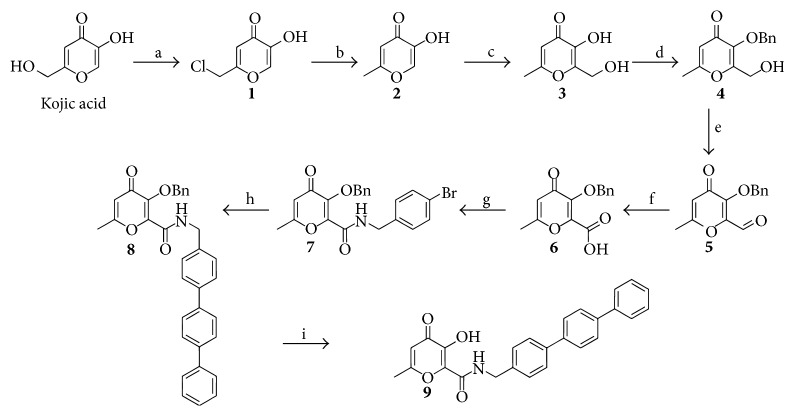
Reagents and conditions: (a) SOCl_2_; (b) H_2_O, Zn, and HCl (aq); (c) NaOH (aq), CH_2_O; (d) NaOH (aq), MeOH, and BnBr; (e) CHCl_3_, Et_3_N, DMSO, and SO_3_-pyridine; (f) acetone/water, sulfamic acid, and NaClO_2_; (g) THF, DCC, NHS, and 4-bromobenzylamine; (h) toluene, K_2_CO_3_ (aq), PPh_3_, Pd(OAc)_2_, and 1,1′-biphenyl-4-boronic acid; (i) MeOH/DCM, Pd/C, and H_2_.

The target compound was synthesized according to the literature with some modifications (Scheme 1). The omission of an aqueous recrystallization step leads to near-quantitative yields of the hydrolytically unstable chlorokojic acid (1) without sacrificing purity. Furthermore, 4-iodobenzylamine could be replaced with 4-bromobenzylamine (g), which is less costly and not sensitive towards light and air [22]. The HCl/acetic acid protocol as written in the original publication [21] did not provide us with the final inhibitor in good yield (some 39% crude yield). Reductive debenzylation (step i) did prove to be a very reliable method to obtain the deprotected molecule. The complete synthetic protocol is included below for the convenience of the reader.

#### 2.1.1. Chemistry: General Procedures

Proton NMR spectra were recorded using a Bruker Avance 300 (300 MHz) device using tetramethylsilane as internal standard for CDCl_3_ and DMSO-d_6_. ^13^C-NMR spectra were recorded using a Bruker Avance 300 (75 MHz) device, using the deuterated solvent as an internal standard. FT-IR spectra were recorded on a Bruker alpha-P. Reagents were purchased from various companies, most notably TCI, Sigma-Aldrich, and Acros. Reagents were not purified prior to use unless specifically noted otherwise.

*(1) Preparation of Compound* ***1*** *(Chlorokojic Acid)*. Kojic acid (TCI, 15 g, 0.105 mol) is placed in a round bottom flask equipped with a magnetic stirring bar. The flask is cooled in an ice bath and thionyl chloride (60 mL) is slowly added after which the ice bath is removed and the mixture is placed under an argon atmosphere. After stirring for 1.5 h a yellow mass is filtered off and washed extensively with petroleum ether. The product was an off-white solid (16.6 g, 98%): ^1^H-NMR (DMSO-d_6_) *δ* 4.6 (s, 2H, Cl-CH2-6), 6.6 (s, 1H, 5-H), and 8.1 (s, 1H, 2-H-) in agreement with the literature [19].

*(2) Preparation of Compound* ***2*** *(Allomaltol)*. Chlorokojic acid** 1** (2.5 g, 0.0156 mol, 1 eq) is dissolved in water (8 mL) and heated to 50°C under an argon atmosphere in a flask equipped with a magnetic stirring bar. Zinc dust (2.1 g, 0.031 mol, 2 eq) is added. An aqueous solution of HCl (4.7 mL, 0.055 mol, 3.5 eq) is slowly added to the reaction mixture under vigorous stirring, during which the temperature is increased to 70°C. After 4 h the excess zinc dust is filtered off while being hot, and the resultant liquid is extracted with dichloromethane (3*∗*20 mL). The combined organic phase is dried over magnesium sulfate and the solvent is removed by evaporation under reduced pressure. The crude material is purified via recrystallization from isopropanol to obtain an off-white solid (1.2 g; 52%): ^1^H-NMR (CDCl_3_) *δ* 2.3 (s, 3H), 6.2 (s, 1H), and 7.8 (s, 1H) in agreement with the literature [19].

*(3) Preparation of Compound* ***3*** *(3-Hydroxy-2-(hydroxymethyl)-6-methyl-4H-pyran-4-one)*. Sodium hydroxide (0.696 g, 0.017 mol, 1.1 eq) is dissolved in water (16 mL). To this solution** 2** (2 g, 0.016 mol, 1 eq) a magnetic stirring bar is added. The solution is stirred for 10 minutes under an argon atmosphere. A 35% aqueous formaldehyde solution (0.36 mL, 0.017 mol, 1.1 eq) is added and the mixture is allowed to stir overnight. After acidification to pH 1 with 35% aqueous hydrochloric acid the mixture is cooled to 2°C and allowed to stand overnight. The crystalline mass is filtered off, washed with 5 mL of water, and dried via lyophilisation. An off-white solid is obtained (1.71 g, 71%): ^1^H-NMR (DMSO-d_6_) *δ* 2.26 (s, 3H), 4.38 (s, 2H), 5.36 (b, s, 1H), 6.21 (s, 1H), and 8.86 (b, s, 1H) in agreement with the literature [19].

*(4) Preparation of Compound* ***4*** *(3-(Benzyloxy)-2-(hydroxymethyl)-6-methyl-4H-pyran-4-one)*. Sodium hydroxide (0.56 g, 8.93 mmol, 1.1 eq) is dissolved in water (2.4 mL) and added to a solution of** 3** (2 g, 8.12 mmol, 1 eq) in methanol (1.2 mL) in a two-necked flask under argon equipped with a reflux condenser, a septum, and a magnetic stirring bar. The mixture is heated to reflux. Benzyl bromide (1.7 mL, 8.93 mmol, 1.1 eq) is slowly added and the mixture is allowed to stir overnight. The mixture is concentrated in vacuo and taken up in dichloromethane (approximately 20 mL). Inorganic salts are filtered off. The mixture is then extracted with 5% aqueous sodium hydroxide (2*∗*20 mL) and brine (1*∗*20 mL). The organic phase is dried with anhydrous sodium sulfate after which the solvent is removed under reduced pressure. The solid is dissolved in as little dichloromethane as possible after which heptane (or petroleum ether) is added until no more precipitation occurs. The precipitate is filtered off and dried to yield a white to off-white powdery solid (2.3 g, 73%): ^1^H-NMR (CDCl_3_) *δ* 2.25 (s, 3), 2.28–4.30 (d, 2H, 6.8 Hz), 5.18 (s, 2H), 6.19 (s, 1H), and 7.37 (s, 5H) in agreement with the literature [19].

*(5) Preparation of Compound* ***5*** *(3-(Benzyloxy)-6-methyl-4-oxo-4H-pyran-2-carbaldehyde)*. Compound** 4** (2 g, 1 eq, 8.18 mmol) is dissolved in 37.8 mL chloroform and 10.2 mL DMSO. To this mixture triethylamine (7 mL, 0.050 mol, 6 eq) and pyridine (0.1 mL) are added. The mixture is stirred in an ice bath under argon using a magnetic stirring bar until a temperature of 3–5°C is reached. Sulfur trioxide-pyridine complex (6.48 g, 0.040 mol, 5 eq) is subsequently added and the mixture is allowed to stir overnight. It is then extracted (2*∗*20 mL water, 1*∗*20 mL brine) and dried over anhydrous sodium sulfate. The solvent is removed under reduced pressure using a rotavapor equipped with a cold trap (dry ice/isopropanol) to obtain the crude product as orange oil. This is diluted with 10 mL diethyl ether and transferred onto a flash column packed with silica/diethyl ether. After eluting with diethyl ether the solvent is removed under reduced pressure using a rotavapor equipped with a cold trap. Remaining volatiles are removed by a stream of nitrogen. A white to yellow crystalline solid is obtained (1.38 g, 69%): ^1^H-NMR (CDCl_3_) *δ* 2.32 (s, 3H), 5.49 (s, 2H), 6.31 (s, 1H), 7.35 (s, 5H), and 9.85 (s, 1H) in agreement with the literature [19]; FT-IR 1690.33 cm^−1^ (C=O aldehyde), 1641.24 cm^−1^ (C=O pyrone), 1613.12 cm^−1^, and 1583.88 cm^−1^ (C=C pyrone).

*(6) Preparation of Compound* ***6*** *(3-(Benzyloxy)-6-methyl-4-oxo-4H-pyran-2-carboxylic acid)*. Compound** 5** (1.57 g, 6.4 mmol, 1 eq) is dissolved in acetone/water (20 mL/20 mL) with a magnetic stirring bar. Sulfamic acid (0.86 g, 8.9 mmol, 1.4 eq) and sodium chlorite (80%, 0.76 g, 6.7 mmol, 1.05 eq) are added and the mixture is allowed to stir for 1 h in an open vessel. Some precipitation may occur. The mixture is subsequently evaporated under reduced pressure to remove the acetone after which the crude product is filtered off and washed with a small amount of ethanol. The product is then dried in vacuo to obtain a stark white powder (1.3 g, 78%): ^1^H-NMR (DMSO-d_6_) *δ* 2.29 (s, 3H), 5.10 (s, 2H), 6.40 (s, 1H), and 7.32–7.45 (m, 5H) in agreement with the literature [19]; FT-IR 1720.37 cm^−1^ (C=O acid), 1625.50 cm^−1^ (C=O pyrone), 1554.48 cm^−1^ and 1496.36 cm^−1^ (C=C pyrone), and MP (precipitated from water/acetone) 180°C.

*(7) Preparation of Compound* ***7*** *(3-(Benzyloxy)-N-(4-bromobenzyl)-6-methyl-4-oxo-4H-pyran-2-carboxamide)*. Compound** 6** (0.1 g, 0.38 mmol, 1 eq) is dissolved in dry tetrahydrofuran (4.16 mL) in a flame-dried round bottom flask equipped with a magnetic stirring bar under an argon atmosphere. N-Hydroxysuccinimide (0.045 g, 0.39 mmol, 1.02 eq) is added and the mixture stirred for 30 min. Dicyclohexylcarbodiimide (0.079 g, 0.38 mmol, 1 eq) is added as a solid and the mixture is stirred for an additional 3 h at RT under an argon atmosphere. Dicyclohexylurea is filtered off and the precipitate is washed with tetrahydrofuran (2 mL). 4-Bromobenzylamine (0.05 mL, 0.385 mmol, 1.01 eq) is added to the filtrate in a dried round bottom flask equipped with a magnetic stirring bar and a reflux condenser under an argon atmosphere. The mixture is heated to 60°C and allowed to stir overnight. The solvent is removed under reduced pressure and the residue taken up in chloroform. This solution is transferred to a chromatography column (silica, eluent chloroform/methanol 1%). After chromatography, the product is obtained as an off-white solid (0.15 g, 91%): ^1^H-NMR (CDCl_3_) *δ* 2.37 (s, 3H), 4.3–4.38 (d, 2H, *J* = 5.6), 5.33 (s, 2H), 6.28 (s, 1H), 6.9–7.5 (m, 9H), and 8.06 (b, s, 1H); FT-IR 3363.82 cm^−1^ (*ν*(NH)), 1677.31 cm^−1^ (*ν*(C=O) pyrone), 1643.58 cm^−1^ (amide I band), 1621.41 cm^−1^ (*ν*(C=C) pyrone), 1584.93 cm^−1^ (*ν*(C=C) pyrone), 1536.21 cm^−1^ (amide II band), and MP (recrystallized from methanol) 118°C.

*(8) Preparation of Compound* ***8*** *(N-([1,1*′*:4*′*,1*′′*-Terphenyl]-4-ylmethyl)-3-(benzyloxy)-6-methyl-4-oxo-4H-pyran-2-carboxamide)*. Compound** 7** (90 mg, 0.21 mmol, 1 eq) is dissolved in toluene in a round bottom flask equipped with a reflux condenser, magnetic stirring bar, and an argon atmosphere. To this mixture are added aqueous potassium carbonate (2 M solution, 10 mL), triphenylphosphine (5.5 mg, 0.021 mmol, 0.1 eq), [1,1′-biphenyl]-4-ylboronic acid (41.5 mg, 0.21 mmol, 1 eq), and palladium acetate (4.6 mg, 0.02 mmol, 0.1 eq) after which the mixture is heated to 135°C with vigorous stirring. The mixture is allowed to stir for 24 h before being extracted with toluene (3*∗*20 mL) and dried over sodium sulfate and the solvent is removed under reduced pressure. The crude product is purified via column chromatography (silica, chloroform/1-2% methanol): ^1^H-NMR (CDCl_3_) *δ* 2.37 (s, 3H), 4.44–4.5 (d, 2H, *J* = 5.7 Hz), 5.34 (s, 2H), 6.29 (s, 1H), 7.10–7.75 (m, 18H), and 8.12 (b, 1H).

*(9) Preparation of Compound* ***9*** *(N-([1,1*′*:4*′*,1*′′*-Terphenyl]-4-ylmethyl)-3-hydroxy-6-methyl-4-oxo-4H-pyran-2-carboxamide)*. Compound** 8** (186.5 mg) is dissolved in dichloromethane/methanol (70/30, 35 mL) in a thick-walled glass vessel. After flushing with argon, the hydrogenation catalyst is added (10% palladium on carbon). The vessel is placed in a shaker-type hydrogenation apparatus and placed under 40 psi hydrogen gas for 20 h after flushing with hydrogen gas to remove traces of oxygen. The catalyst is filtered off and the filtrate washed with 10 mL dichloromethane/methanol (50/50). The combined organic phase is evaporated in vacuo to yield an orange solid (138.5 mg, 90%): ^1^H-NMR (CDCl_3_) 2.24 (s, 3H), 4.6 (s, 2H), 6.16 (s, 1H), and 7–7.8 (m, 13H); FT-IR 2339.5 cm^−1^ (OH), 1633.1 cm^−1^ (amide I), 1591.8 cm^−1^ (C=O pyrone), 1537.5 cm^−1^ (amide II), 1483.9 cm^−1^ (C=C pyrone), and MP (recrystallized from toluene) 253–270°C.

### 2.2. Recombinant MMP Assays

The specificity of the synthesized inhibitor was determined* in vitro* against a panel of ten human recombinant MMP catalytic domains using a fluorescent assay kit (BML-AK016, Enzo Life Sciences). Briefly, the compound was dissolved in dimethyl sulfoxide (DMSO) and further diluted in assay buffer (50 mM HEPES, 10 mM CaCl_2_, 0.05% Brij-35, and pH 7.5). Ten MMPs (MMP-1, MMP-2, MMP-3, MMP-7, MMP-8, MMP-9, MMP-10, MMP-12, MMP-13, and MMP-14) were individually incubated with varying concentrations of inhibitor for one hour at 37°C, followed by addition of a quenched fluorogenic substrate (OmniMMP fluorogenic substrate Mca-Pro-Leu-Gly-Leu-Dpa-Ala-Arg-NH2 [Mca is (7-methoxycoumarin-4-yl)-acetyl; Dpa is N-3-(2,4-dinitrophenyl)-L-*α*-*β*-diaminopropionyl], 4 *μ*M in assay). The assays were performed in black, clear bottom, NBS Greiner 96-well plates (655906, Greiner Bio-One) and fluorescence (*λ*_ex_ = 325 nm, *λ*_em_ = 405 nm) was measured at 60 sec intervals for 30 min with a FlexStation II microplate reader running SoftMax Pro 5.3 (Molecular Devices). After each fluorescent measurement the well plates were shaken for 2 sec to agitate the reagents. In each experiment we included a blank, a substrate control (substrate only in assay buffer), a positive control (NNGH, 1.3 *μ*M final concentration), and negative control (only substrate and inhibitor in assay buffer). The latter was implemented to exclude possible interference of the inhibitor with the substrate. To confirm the potency against MMP-3 and MMP-12, separate assays were performed using human recombinant MMP-3 (72006, AnaSpec) and MMP-12 catalytic domains (55525-1, AnaSpec) and quenched fluorescent substrate QXL 520-Pro-Leu-Ala-Tyr-Trp-Ala-Arg-Lys(5- FAM)-NH2 (*λ*_ex_ = 485 nm, *λ*_em_ = 538 nm, 60577-01, AnaSpec). The IC_50_ values were determined in a single experimental run in duplicate. Data analysis and curve fitting for determination of IC_50_ values were performed in GraphPad Prism 6.

### 2.3. Animals

Female C57BL/6J (8–10 weeks old) mice were purchased from Janvier Labs (France) and MMP-12 knockout mice (MMP-12^−/−^) (in C57BL/6J background together with littermate MMP-12^+/+^ controls) were obtained from Dr. Steven Shapiro (University of Pittsburgh, USA). C57BL/6 and MMP-12^−/−^ mice were housed with 4–6 mice/cage with* ad libitum* access to food and water and with a 14-hour light/10-hour dark cycle in a specific pathogen-free animal facility and conventional facility, respectively. All experiments were approved by the Ethics Committee of the Faculty of Sciences of Ghent University.

### 2.4. Endotoxemia Model and MMP-12i Treatment

Endotoxemia was induced by intraperitoneal (i.p.) injection of lipopolysaccharide (LPS) from* Salmonella enterica* serotype abortus equi (Sigma) dissolved in PBS. The dose was 200 *μ*g/20 g body weight (the LD_100_ dose for C57BL/6 mice). Control animals received i.p. injections of PBS. Rectal temperature was measured periodically after challenge. 100 *μ*g MMP-12i (1 *μ*g/*μ*L) or vehicle control was administered intraperitoneally at different time points: 15 min before and 3 and 6 hours after LPS administration.

### 2.5. Gene Expression Analysis

For isolation of total RNA from* in vivo* choroid plexus samples, mice were anesthetized with ketamine/xylazine and perfused with PBS supplemented with bromophenol blue. Brains were dissected from the skull and choroid plexus from the third and fourth ventricles were dissected under a dissection microscope. Total RNA was isolated at different time points after LPS injection with the RNeasy kit (Qiagen). RNA concentration and purity were determined spectrophotometrically using the NanoDrop ND-1000 (NanoDrop Technologies). cDNA was made by using iScript cDNA Synthesis Kit (Bio-Rad) with 500 ng starting material and QPCR was done using the SensiFAST SYBR No-ROX Kit (Bioline) on the Light Cycler 480 system (Roche). Expression levels of* Mmp-12* were normalized to the expression of the two most stable housekeeping genes,* Ubc* and* Gapdh,* which were determined using the geNorm Housekeeping Gene (HKG) Selection Software [23]. Primer sequences can be found in Table 1.

### 2.6. Blood-CSF Barrier Permeability Leakage* In Vivo*

One hour before CSF isolation, mice were injected i.v. with 75 mg/kg body weight of FITC-labeled dextran (4 kDa, Sigma). They were perfused with 0.9% saline to remove all labeled dextran in the circulation. CSF was obtained using the cisterna magna puncture method as described previously [24]. In brief, borosilicate glass capillary tubes (B100-75-15, Sutter Instruments) were used to pull needles on the Sutter P-87 flaming micropipette puller (pressure 330 Pa, heat index 300). Before sampling CSF, mice were sedated with ketamine/xylazine. The incision site was sterilized with 70% ethanol, and cisterna magna was exposed by cutting skin and muscle tissue on the posterior side of the skull. The head of the mouse was placed at an angle of 135 degrees and CSF was collected by inserting the trimmed needle into the fourth ventricle by piercing the cisterna magna. A CSF sample of 2 *μ*L was diluted 25-fold in sterile PBS and leakage into this brain compartment was determined by measurement of fluorescence with *λ*_ex_/*λ*_em_ = 488/520 nm.

### 2.7. Blood-CSF Barrier Permeability* In Vitro*

Culturing primary mouse choroid plexus epithelial cells was done as described [25]. In brief, 2–9-day-old pups were decapitated and brains were isolated. Choroid plexuses from all four ventricles were isolated under a dissection microscope. The cells were dissociated enzymatically by incubating them for 5–7 min with pronase (isolated from* Streptomyces griseus*, Sigma). Digestion was stopped by adding an excess of HBSS buffer and the cells were washed twice with HBSS. The cell pellet was resuspended in DMEM-F12 culture medium and plated on laminin-coated plates. Two days later, they were shifted to DMEM-F12 containing cytosine arabinoside (Ara-C) to eliminate growth of fibroblasts. Cultures were maintained at 37°C in 5% CO_2_. Purity of the cultures was confirmed by checking the levels of transthyretin (TTR) both by qPCR and by immunostaining. Primary CPE cells retained the epithelial and the barrier properties which were confirmed by staining for E-cadherin, ZO-1, and occludin. The electric cell-substrate impedance (ECIS) technique (Applied Biophysics) was used to monitor barrier properties as described before [26]. 250.000 cells per well were seeded onto laminin-coated 8W10E array plates (Applied Biophysics) and the array holder was placed in a standard cell culture incubator (37°C, 100% humidity, and 5% CO_2_). Array plates were connected to the instrument and multifrequency measurements were started. After ~16 hours, a stable impedance at low and high frequency was obtained and cells were incubated with vehicle or 1 *μ*M MMP-12i, followed 1 hour later by vehicle or LPS (1000 ng/mL).

## 3. Results and Discussion

### 3.1. Inhibitor Specificity

**Figure 1:**
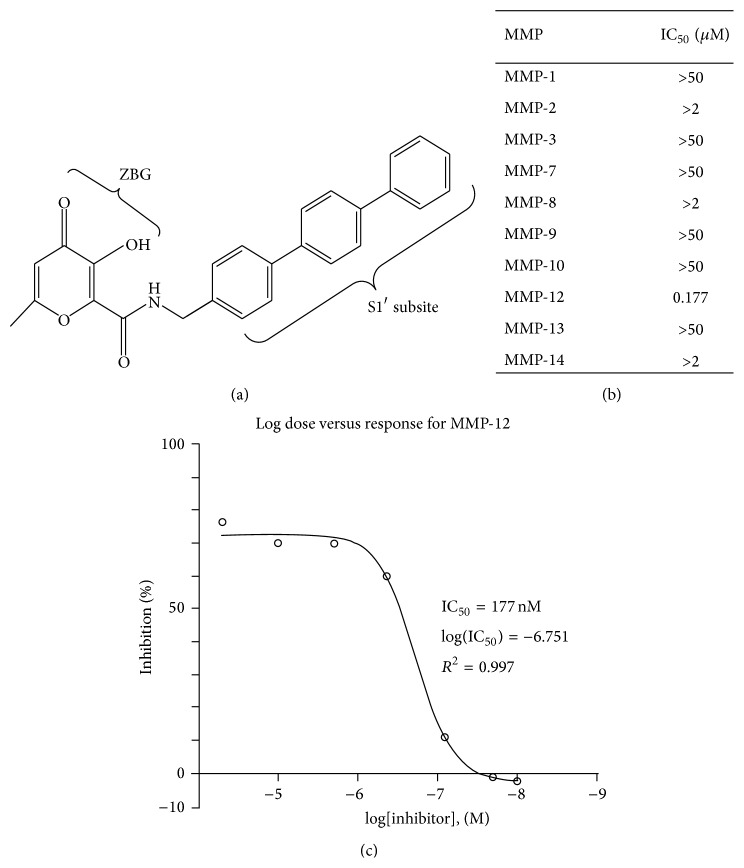
Chemical structure and specificity of the synthesized MMP inhibitor. (a) The target compound consists of a Zn^2+^-binding group (ZBG) and a terphenyl which resides in the deep S1′ pocket of the MMP catalytic domain [21]. (b) List of* in vitro* determined IC_50_ values (*μ*M) which indicates that the inhibitor is very specific towards MMP-12 with an IC_50_ in the nanomolar range. (c) Fitted dose-response curve relation to MMP-12 used to calculate the IC_50_.

Compound** 9** (Figure 1(a)) was assessed for its inhibitory properties towards an array of ten different MMPs using a fluorescent* in vitro* assay. For all tested MMPs, the compound is poorly potent against MMP-1, MMP-3, MMP-7, MMP-9, MMP-10, and MMP-13, reflecting IC_50_ values higher than 50 *μ*M (Figure 1(b)). The compound displays slightly better inhibitory activity towards MMP-2, MMP-8, and MMP-14 as the apparent IC_50_ values are situated between 2 *μ*M and 50 *μ*M. But most importantly towards MMP-12 the compound reveals very potent inhibitory activity. The initial screen revealed an apparent IC_50_ value for MMP-12 below 2 *μ*M and when performing a detailed dose-response experiment using a more specific substrate for MMP-12 (Substrate XIII, AnaSpec) a nonlinear regression analysis indicated that at higher inhibitor concentrations an inhibitory plateau (72 ± 1.75%) is reached with a relative IC_50_ of 177 nM (Figure 1(c)). Although the use of a different substrate can potentially influence the kinetic readout, a comparison between the fluorescent* in vitro* assay kit and the use of Substrate XIII resulted in similar inhibitor potencies (data not shown), which justifies the use of the more specific substrate for MMP-12. Taken together, these data indicate that the synthesized compound is a potent and fairly specific inhibitor of MMP-12 and therefore from Section 3.2 onwards it will be referred to as MMP-12i. The discrepancy between Puerta et al. [21] and this study regarding the inhibitory selectivity towards MMP-12 over MMP-3 could in part be explained by the strong pH-dependence of inhibitor potency for human MMP-3 [27]. Both MMP-3 and MMP-12 have a deep pocket S1′ subsite [28] in which the terphenyl can reside [21]. However, MMP-3 is uniquely characterized by a sharp optimum at pH 6 for efficient catalysis and inhibition and this is drastically reduced at neutral pH by structural changes in the S1′ pocket and ionization of inhibitor residues [27]. In contrast to the study of Puerta and colleagues, the compound was tested in physiologically relevant pH 7.5 to accommodate a large array of MMPs. Importantly, the pH-dependence of inhibitor-MMP-3 interactions typically gives rise to a change in IC_50_ values not larger than one order of magnitude [27] as opposed to the three orders of magnitude we established here, so other factors might be at play. Because of this and the observed potency towards MMP-12 we further validated the biological relevance of the compound in the context of MMP-12.

### 3.2. *In Vitro* and* In Vivo* Effect of MMP-12 Inhibition

**Figure 2:**
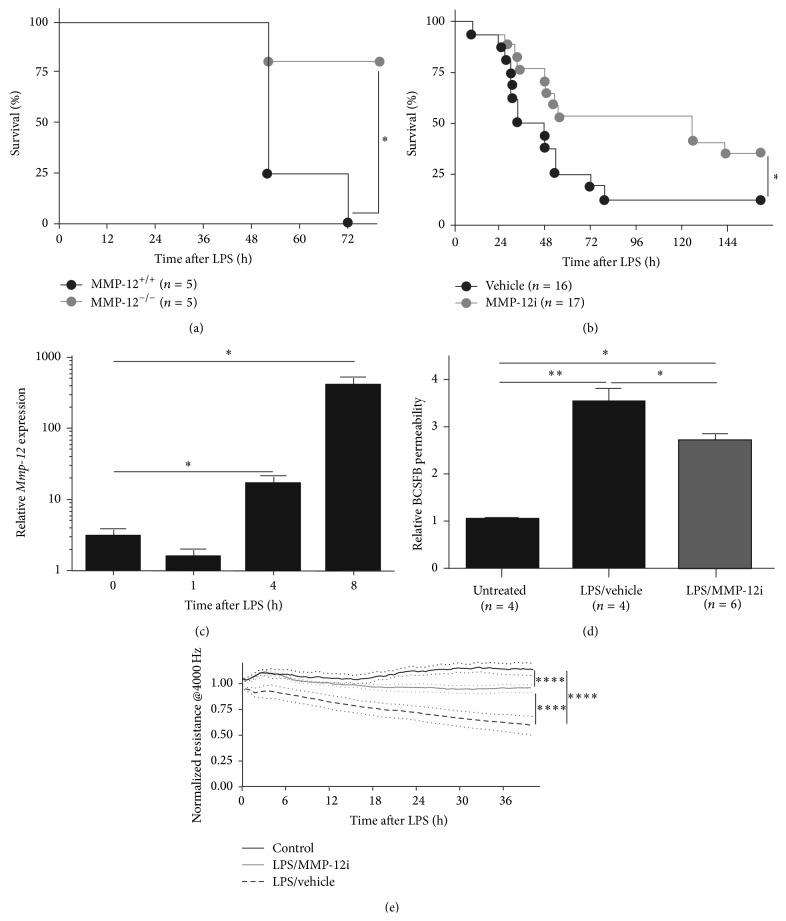
*In vivo* and* in vitro* effect of MMP-12 inhibition in endotoxemia. (a) Survival in function of time of MMP-12^+/+^ (black) and MMP-12^−/−^ (grey) mice following i.p. challenge with a lethal dose of LPS. (b) Survival in function of time of C57BL/6 mice injected with vehicle (black) or MMP-12i (grey) following i.p. challenge with a lethal dose of LPS. (c) Relative* Mmp-12* expression in choroid plexus tissue isolated before and 1, 4, and 8 hours after LPS challenge (*n* = 3-4). (d) Relative permeability of the blood-CSF barrier determined by measuring leakage of fluorescently labeled dextran (4 kDa) from the blood into the CSF 8 hours after LPS challenge in vehicle (black) and MMP-12i (grey) treated wild type mice, compared to PBS injected wild type mice. (e) Normalized resistance measured at low frequency using the ECIS instrument of primary choroid plexus epithelial cells incubated with vehicle (black), 1000 ng/mL LPS (black dotted line), and 1000 ng/mL LPS pretreated with 1 *μ*M MMP-12 inhibitor (grey) (*n* = 3). Data are presented as means ± standard error of mean (sem). Survival curves were compared using a log-rank test. Other data were analyzed by Student's* t*-test. ^*∗*^  0.01 ≤ *P* < 0.05; ^*∗∗*^  0.0001 ≤ *P* < 0.001; ^*∗∗∗∗*^ *P*  < 0.0001.

Endotoxemia, that is, systemic administration of lipopolysaccharide (LPS), induces systemic inflammation and eventually lethality in mice. We have previously shown that mice can be protected from LPS-induced lethality by administration of a broad spectrum MMP inhibitor BB-94 [9] and several MMPs appeared to play a role in the LPS-induced lethality [8, 11–13]. Here, we studied the effect of MMP-12 deficiency on the LPS sensitivity by using MMP-12^−/−^ mice. As shown in Figure 2(a), MMP-12^−/−^ mice display a partial protection to an LD_100_ dose of LPS, indicating that MMP-12 plays a detrimental role in the LPS-induced lethality. To verify this, we treated wild type mice with the MMP-12i and challenged them with LPS (Figure 2(b)). MMP-12i treatment resulted in a significant reduction in LPS-induced lethality compared with vehicle treated mice, which again demonstrates that the compound most likely targets MMP-12. Systemic inflammation is known to be associated with loss of blood-brain barrier integrity and this has been correlated with lethality [29]. Next to the most studied endothelial blood-brain barrier (BBB), we have previously shown that the blood-cerebrospinal fluid barrier (BCSFB) which is formed by the choroid plexus epithelium (CPE) is also affected in response to systemic LPS administration [9]. mRNA expression analysis of choroid plexus tissue revealed 500-fold upregulation of* Mmp-12* upon systemic LPS administration (Figure 2(c)), associated with an increase in BCSFB permeability (Figure 2(d)). Interestingly, the loss of BCSFB permeability was significantly reduced when endotoxic mice were treated with our MMP-12i (Figure 2(d)). Finally, we analyzed the effect of the MMP-12i on the BCSFB* in vitro*. Therefore, primary CPE cells were isolated and plated onto electrical cell impedance sensing (ECIS) arrays containing gold electrodes. ECIS is a real-time, label-free, impedance-based method used to study the barrier properties of cells grown in culture. After reaching confluency, cells were incubated with LPS or LPS supplemented with MMP-12i and compared to untreated cells. LPS resulted in a drop in normalized resistance compared to control and this was significantly reduced in the presence of MMP-12i (Figure 2(e)). These data clearly show that MMP-12 plays a role in the observed LPS-induced loss of BCSFB integrity.

## 4. Conclusion

In summary, we report the synthesis and biological evaluation of a hydroxypyrone-based MMPi with excellent potency and specificity towards MMP-12. Both* in vivo* and* in vitro* studies on the effects of the target compound on endotoxemia and BCSFB integrity clearly indicate a role for MMP-12 in these inflammatory processes and establish the use of this compound in a biological context.

## Figures and Tables

**Table 1:** Primer sequences used for gene expression analysis.

Primer	Sequence
*Ubc* forward	AGGTCAAACAGGAAGACAGACGTA
*Ubc* reverse	TCACACCCAAGAACAAGCACA
*Gapdh* forward	TGAAGCAGGCATCTGAGGG
*Gapdh* reverse	CGAAGGTGGAAGAGTGGGAG
*Mmp-12* forward	CTGCTCCCATGAATGACAGTG
*Mmp-12* reverse	AGTTGCTTCTAGCCCAAAGAAC

Ubc, ubiquitin C; Gapdh, glyceraldehyde 3-phosphate dehydrogenase.
